# PSMB1 Negatively Regulates the Innate Antiviral Immunity by Facilitating Degradation of IKK-ε

**DOI:** 10.3390/v11020099

**Published:** 2019-01-24

**Authors:** Fangyi Wu, Zhenmin Niu, Bin Zhou, Pengcheng Li, Feng Qian

**Affiliations:** 1 Ministry of Education Key Laboratory of Contemporary Anthropology, Human Phenome Institute, School of Life Sciences, Fudan University, Shanghai 200438, China; wufangyi_fd@163.com (F.W.); zhoubin1999fd@163.com (B.Z.); lipengcheng_fdu@163.com (P.L.); 2 Department of Genetics, Shanghai-MOST Key Laboratory of Health and Disease Genomics, Chinese National Human Genome Center, Shanghai Academy of Science and Technology, Shanghai 201203, China; niuzhm@chgc.sh.cn

**Keywords:** PSMB1, IKK-ε, innate immunity, cytokine, RIG-I, TLR3

## Abstract

Proteasome is a large protein complex, which degrades most intracellular proteins. It regulates numerous cellular processes, including the removal of misfolded or unfolded proteins, cell cycle control, and regulation of apoptosis. However, the function of proteasome subunits in viral immunity has not been well characterized. In this study, we identified PSMB1, a member of the proteasome β subunits (PSMB) family, as a negative regulator of innate immune responses during viral infection. Knockdown of *PSMB1* enhanced the RNA virus-induced cytokine and chemokine production. Overexpression of PSMB1 abolished virus-induced activation of the interferon-stimulated response element (ISRE) and interferon beta (IFNβ) promoters. Mechanistically, PSMB1 inhibited the activation of RIG-I-like receptor (RLR) and Toll-like receptor 3 (TLR3) signaling pathways. PSMB1 was induced after viral infection and its interaction with IKK-ε promoted degradation of IKK-ε through the ubiquitin-proteasome system. Collectively, our study demonstrates PSMB1 is an important regulator of innate immune signaling.

## 1. Introduction

The innate immune system can rapidly detect invading viruses and establish an antiviral state [1]. Following viral infection, viral nucleic acids can be recognized by pattern recognition receptors (PRRs) to initiate the cellular antiviral responses [2]. Toll-like receptors (TLRs) and RIG-I like receptors (RLRs) are the main PRRs that sense distinct types of RNA viruses [3,4]. Upon recognition, PRRs trigger the activation of a downstream signaling pathway, leading to the production of type I IFNs and proinflammatory cytokines [5]. Secreted type I interferons (IFNs) further activate the Janus kinase (JAK)/Signal transducer and activator of transcription (STAT) pathway, trigger the production of interferon stimulated genes (ISGs) and suppress viral replication and infection [6]. In addition to the antiviral activity, excessive production of type I IFNs is associated with inflammatory diseases and autoimmune diseases. Therefore, precise regulation of type I IFN production is a significant issue.

IκB kinase ε (IKK-ε) has an essential role in the activation of interferon signaling. Upon viral infection, Toll-like receptor 3 (TLR3) and RIG-I recruit their adaptor protein (TIR-domain-containing adapter-inducing interferon-β, TRIF for TLR3 and mitochondrial antiviral signaling protein, MAVS for RIG-I) [7]. These adaptors further interact with TNF receptor associated factor 3 (TRAF3) and result in IKK-ε auto-phosphorylation-mediated activation [8]. Activated IKK-ε directly phosphorylates IRF3/IRF7 and induces the production of type I IFN [9]. To avoid excessive IFN production, IKK-ε activity must be tightly regulated.

The proteasome recognizes the poly-ubiquitinated substrates and degrades intracellular proteins [10]. The ubiquitin-proteasome system (UPS) is an essential mechanism in the regulation of signal transduction and inflammatory responses [11]. The dysregulations of the UPS contribute to the pathogenesis of neurodegenerative disorders and autoimmune diseases [12,13]. Proteasomes consist of a 20S core complex and a 19S regulatory complex. The 20S core particle is composed of two outer α-rings and two inner β-rings. The proteasome β subunits (PSMBs) are the components of the inner β-ring and active sites of 20S with proteolytic specificities [14]. However, the role of PSMB subunits in innate immunity has not been well characterized. 

In this study, we demonstrate that PSMB1 negatively regulates the innate immune responses during viral infection. The silencing of *PSMB1* enhances the production of antiviral cytokines, resulting in attenuated viral replication in the virus-infected cells. Overexpression of PSMB1 inhibits RIG-I- and TLR3-mediated type I IFN responses. Our results describe a previously unknown role of PSMB1 in the regulation of immune responses.

## 2. Materials and Methods

### 2.1. Cell Lines and Reagents

THP-1, HEK293T, A549, and Vero cells were obtained from Type Culture Collection of the Chinese Academy of Science. The cells were cultured at 37 °C under 5% CO_2_ in RPMI 1640 or DMEM medium supplemented with 10% fetal bovine serum and antibiotics (100 units/mL penicillin and 100 μg/mL streptomycin, Invitrogen, Carlsbad, CA, USA). To generate stable *PSMB1* knockdown cell lines, *PSMB1*-specific shRNA (TRCN0000003898, TRCN0000003900, Broad Institute, Cambridge, MA, USA) were retrovirally transfected into HEK293T cells [15]. To select transduced cells, puromycin (2ug/mL) was added to the media. Protein knockdown efficiency was analyzed by immunoblot analysis.

Low molecular weight (LMW) and high molecular weight (HMW) Poly I:C were purchased from Invivogen (San Diego, CA, USA). The antibody specific to IKK-ε was from Cell Signaling Technology (Danvers, MA, USA). Anti-HA, anti-Flag, anti-PSMB1 and anti-GAPDH antibodies were from Proteintech (WuHan, China). HRP-conjugated secondary antibodies were from Sungene Biotechnology (Beijing, China). Cycloheximide (CHX) and MG132 were purchased from Sigma-Aldrich (St.Louis, MO, USA). 

### 2.2. Viruses

Influenza A virus (IAV, PR8 strain) was a kind gift from Dr. Yang Li (Fudan University, Shanghai, China). Vesicular stomatitis virus with green fluorescent protein (GFP) (VSV-GFP) was provided by Dr. Guang Yang (Jinan University, Guangzhou, China). Virus titers were quantified by 50% tissue culture infective dose (TCID50) assay. 

### 2.3. Dual-Luciferase Reporter Assay

HEK293T cells were transfected with indicated expression plasmids along with luciferase reporter plasmids pGL3-IFNβ, pISRE-Luc, pNF-kB-Luc (Stratagene, La Jolla, CA, USA), or pRL-TK (Promega, Fitchburg, WI, USA) using Hieff TransTM transfection reagent (Yeasen, Shanghai, China) for 24 h in 24-well plates. Then, cells were treated with Poly I:C or infected with virus. Luciferase activities were measured with Dual-Luciferase Reporter Assay System (Transgene, Beijing, China).

### 2.4. Quantitative Polymerase Chain Reaction (qPCR) Analysis

Total RNA was extracted with TRI Reagent (Sigma, St.Louis, MO, USA) and cDNA synthesis was performed by the Reverse Transcription Reagent Kit (abm, Vancouver, BC, Canada) according to the manufacturer’s instructions. CFX96 System (Bio-Rad, Berkeley, CA, USA) and SYBR Green qPCR Master Mix (Biotools, Houston, TX, USA) were used for quantitative real-time PCR analysis. Data were normalized to *β-ACTIN* expression in each sample. The specific primers used for qPCR were as follows: *PSMB1* (forward: 5′-CTA CAA TCC TGT ATT CAA GGC GC-3′, reverse: 5′-TCC AGC CTT GAA GGA GTC TCT C-3′), *IKBKE* (forward: 5′-GGC TAC AAC GAG GAG CAG ATT C-3′, reverse: 5′- GGA CGC TTG ATA CTT CTG CAC G-3′), *IFNB1* (forward: 5′-CTT GGA TTC CTA CAA AGA AGC AGC-3′, reverse: 5′-TCC TCC TTC TGG AAC TGC TGC A-3′), *CCL5* (forward: 5′-CCT GCT GCT TTG CCT ACA TTG C-3′, reverse: 5′- ACA CAC TTG GCG GTT CTT TCG G-3′), *IFIT1* (forward: 5′-GCC TTG CTG AAG TGT GGA GGA A-3′, reverse: 5′- ATC CAG GCG ATA GGC AGA GAT C-3′), *TNFA* (forward: 5′-CTC TTC TGC CTG CTG CAC TTT G-3′, reverse: 5′-ATG GGC TAC AGG CTT GTC ACT C-3′) and *β-ACTIN* (forward: 5′-AGA TCA TGT TTG AGA CCT TCA ACA C -3′, reverse: 5′-GGA GCA ATG ATC TTG ATC TTC ATT G -3′) 

### 2.5. Cytokine ELISA Measurements and Type I IFN Bioassays 

Cell supernatants were harvested from virus infected cells. The concentration of TNF-α was quantified by the ELISA MAXTM Deluxe kit according to the manufacturer’s instructions (Biolegend, San Diego, CA, USA). Type I IFNs were measured using a 2fTGH cell line stably expressing interferon-stimulated response element (ISRE)-Luc reporter [16]. In brief, supernatants from infected and uninfected cells were incubated with 2fTGH-ISRE reporter cells for 6 h. Cells were lysed and subjected to luciferase quantification. A serial dilution of human IFNβ (Peprotech, Rocky Hill, NJ, USA) was included as standards.

### 2.6. Immunoblot Analysis and Immunoprecipitation (IP) 

For immunoblot analysis, cells were lysed with RIPA III lysis buffer containing protease inhibitor cocktail (Biotechwell, Shanghai, China). Equal amounts of extracts were separated by SDS-PAGE and then transferred onto polyvinylidene fluoride (PVDF) membranes (Bio-rad, Berkeley, CA, USA). Immunoblots were probed with antibody as described and developed using enhanced chemiluminescence (ECL) reagents (NCM Biotech, Suzhou, China). 

For immunoprecipitation, whole cell extracts were incubated with protein A/G agarose beads (Santa Cruz, CA, USA) together with specific antibody. After 6 h of incubation, beads were washed five times with lysis buffer. Proteins were eluted by boiling for 5 min in SDS sample buffer. 

### 2.7. Statistical Analysis

Statistical significance was determined by an unpaired Student’s *t*-test with GraphPad Prism (San Diego, CA, USA). Data were presented as the mean ± SEM of three independent experiments. Values of *p* < 0.05 were considered statistically significant. 

## 3. Results

### 3.1. PSMB1 Is Involved in Cellular Antiviral Responses

**Figure 1:**
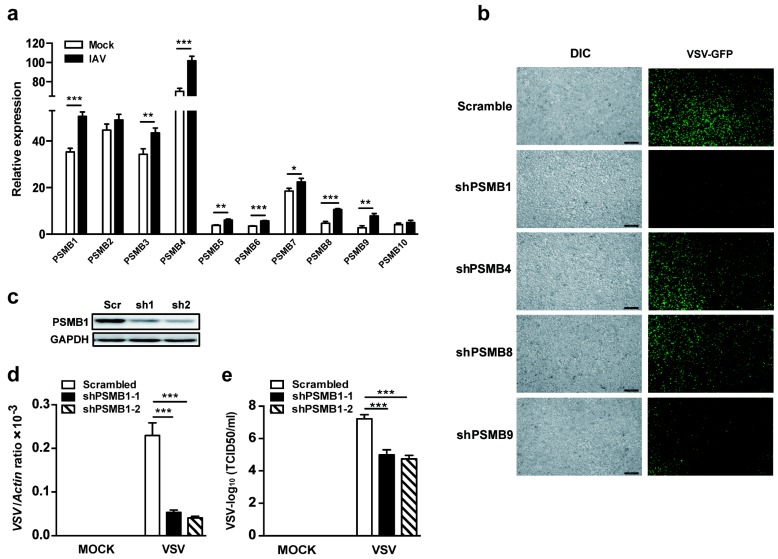
PSMB1 (proteasome β subunit 1) is involved in cellular antiviral responses. (**a**) Quantification of *PSMB* mRNA levels by qPCR in THP-1 cells after influenza A virus (IAV, PR8 strain) infection for 24 h, multiplicity of infection (MOI) = 1. (**b**) Microscopic images of vesicular stomatitis virus (VSV)-GFP-infected HEK293T cells expressing either scrambled shRNA or indicated *PSMB*-specific shRNA. Images were acquired with a Leica fluorescence microscope. Scale bars, 100 μm. (**c**) Immunoblot analysis of PSMB1 expression in HEK293T cells stably expressing shRNA against *PSMB1*. (d, e) Quantification of VSV loads by qPCR (**d**) and VSV titers by TCID50 (**e**) from HEK293T cells stably expressing either scrambled shRNA or *PSMB1*-targeting shRNA after VSV infection for 12 h (MOI = 0.1). Data shown are the mean ± SEM; * *p* < 0.05, ** *p* < 0.01, *** *p* < 0.001. Representative results are from at least three independent experiments.

To explore the potential role of the PSMB family in cellular antiviral responses, we examined the expression pattern of the PSMB family in human THP-1 monocytes upon virus infection. The mRNA expression levels of *PSMBs* were detected by qPCR. Except for *PSMB2* and *PSMB10*, all other *PSMBs* were upregulated after influenza A virus (IAV, PR8 strain) infection of THP-1 cells (Figure 1a). We then silenced each of the top four upregulated *PSMBs* (*PSMB1, PSMB4, PSMB8* and *PSMB9*, fold change >1.5) using shRNA in HEK293T cells and examined the viral replication in *PSMB*-silenced cells after VSV-expressing GFP (VSV-GFP) infection. Results showed that VSV replication in terms of GFP intensity was significantly reduced in *PSMB1*-silenced cells (Figure 1b). To further confirm this, we used two shRNAs that targeted different sites of *PSMB1* and generated *PSMB1*-silenced HEK293T cells. Endogenous *PSMB1* was silenced efficiently, as quantified by immunoblot analysis (Figure 1c). We measured the replication of VSV in *PSMB1*-silenced cells. The results demonstrated that both viral RNA levels measured by qPCR and the virus titers determined by TCID50 showed that silencing *PSMB1* attenuated the viral replication in HEK293T cells (Figure 1d,e). These data suggest that PSMB1 is involved in cellular antiviral responses.

### 3.2. PSMB1 Negatively Regulates RNA Virus-Induced Innate Immune Responses

**Figure 2:**
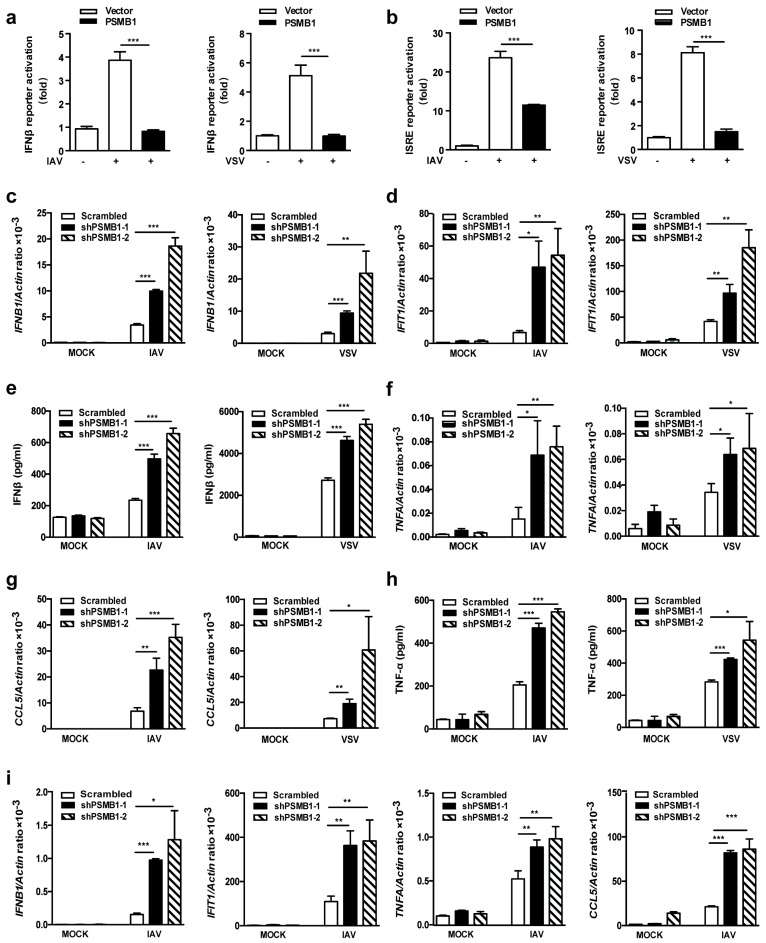
PSMB1 negatively regulates RNA virus-induced innate immune responses. (**a**,**b**) Quantification of IFNβ and ISRE promoter activities in HEK293T cells transfected with either empty vector or PSMB1 plasmid after IAV or VSV infection. (**c**–**e**) Quantification of *IFNB1* and *IFIT1* mRNA levels by qPCR (c, d) and secreted interferon beta (IFNβ) protein levels (e) from HEK293T cells stably expressing either scrambled shRNA or *PSMB1*-targeting shRNA after IAV infection (MOI = 1) for 24 h or VSV infection (MOI = 0.1) for 12 h. (**f**–**h**) Quantification of *TNFA* and *CCL5* mRNA levels by qPCR (f, g) and secreted tumor necrosis factor alpha (TNF-α) levels by ELISA (h) from HEK293T cells stably expressing either scrambled shRNA or *PSMB1*-targeting shRNA after IAV infection (MOI = 1) for 24 h or VSV infection (MOI = 0.1) for 12 h. (**i**) Quantification of indicated cytokine and chemokine mRNA levels by qPCR from A549 cells stably expressing either scrambled shRNA or *PSMB1*-targeting shRNA after IAV infection (MOI = 1) for 24 h. Data shown are the mean ± SEM; * *p* < 0.05, ** *p* < 0.01, *** *p* < 0.001. Representative results are from at least three independent experiments.

Induction of type I IFN is a key step of antiviral innate immune responses. We evaluated the effect of PSMB1 on type I IFN responses using luciferase reporter assay. Overexpression of PSMB1 significantly inhibited IAV- and VSV-induced IFNβ-promoter activation (Figure 2a). In addition, virus induced ISRE activation was also reduced by PSMB1 (Figure 2b). We further quantified mRNA expression levels of *IFNB1* and interferon induced protein with tetratricopeptide repeats 1(*IFIT1)* in *PSMB1*-silenced cells by qPCR. *PSMB1* knockdown significantly increased the transcription level of *IFNB1* and *IFIT1* after IAV and VSV infection (Figure 2c,d). Consistent with the transcriptional data, the virus-induced IFNβ protein level was also increased in the *PSMB1*-silenced cells (Figure 2e).

Virus infections trigger the immune responses, including the expression of proinflammatory cytokines and chemokines [17]. We also measured the expression of proinflammatory cytokine *TNFA* and chemokine *CCL5* in *PSMB1*-silenced cells. In agreement with the *IFNB1* findings, silencing of *PSMB1* promoted the mRNA expression levels of *TNFA* and *CCL5* after infection with IAV and VSV (Figure 2f,g). The protein level of TNF-α was also increased in *PSMB1*-silenced cells (Figure 2h). Consistent with these results, the mRNA expression levels of *IFNB1*, *IFIT1*, *TNFA,* and *CCL5* in *PSMB1*-silenced A549 cells were significantly increased after IAV infection (Figure 2i). These results suggest that PSMB1 is involved in the regulation of RNA virus-induced innate immune responses.

### 3.3. PSMB1 Inhibits RLR and TLR3 Signaling

**Figure 3:**
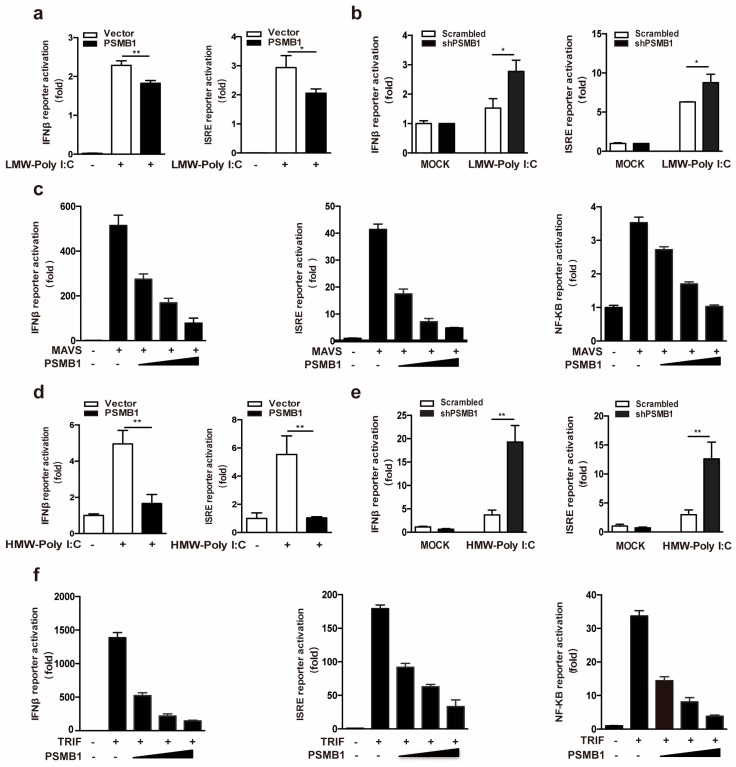
PSMB1 Inhibits RIG-I-like Receptor (RLR) and Toll-like receptor 3 (TLR3) signaling. (**a**) Quantification of IFNβ and ISRE promoter activities of HEK293T cells transfected with either an empty vector or PSMB1 plasmid and treated with LMW Poly I:C. (**b**) Quantification of IFNβ and ISRE promoter activities in scrambled shRNA or *PSMB1* shRNA treated HEK293T cells after LMW Poly I:C stimulation. (**c**) Quantification of IFNβ, ISRE, and NF-κB promoter activities of HEK293T cells expressing mitochondrial antiviral signaling protein (MAVS) together with increasing amounts of PSMB1. (**d**) Quantification of IFNβ and ISRE promoter activities of HEK293T-TLR3 cells transfected with either an empty vector or PSMB1 plasmid and treated with HMW Poly I:C. (**e**) Quantification of IFNβ and ISRE promoter activities in scrambled shRNA or *PSMB1* shRNA treated HEK293T-TLR3 cells after HMW Poly I:C stimulation. (**f**) Quantification of IFNβ, ISRE, and NF-κB promoter activities of HEK293T cells expressing TRIF, together with increasing amounts of PSMB1. Data shown are the mean ± SEM; * *p* < 0.05, ** *p* < 0.01. Representative results are from at least three independent experiments.

RLRs recognize cytoplasmic viral RNA and initiate antiviral innate immune responses [18]. To investigate whether PSMB1 is involved in the regulation of RLR signaling, we evaluated the effect of PSMB1 on RLR-dependent IFNβ activation. Overexpression of PSMB1 significantly inhibited the activation of IFNβ and ISRE reporter cells in HEK293T cells transfected with synthetic analogs of viral RNAs, low-molecular-weight (LMW) Poly I:C (RIG-I agonist) (Figure 3a). In agreement with this finding, *PSMB1* knockdown enhanced the IFNβ and ISRE reporter cell luciferase activity after LMW Poly I:C transfection (Figure 3b). Besides, we assessed the impact of PSMB1 on key components of RLR signaling in their activation of IFNβ, ISRE, and NF-κB promoters. HEK293T cells were transfected with the RLR signaling adaptor molecule MAVS and increasing amounts of PSMB1. The reporter assay results showed that PSMB1 inhibited the MAVS-induced IFNβ, ISRE, and NF-κB promoter activation in a dose-dependent manner (Figure 3c).

TLR3 recognizes the viral RNA in the endosome and triggers a series of signaling events to activate the antiviral responses [19]. We next examined whether PSMB1 regulates the TLR3 signaling pathway. The effects of PSMB1 on TLR3-dependent IFNβ activation were observed. As shown in Figure 3d, PSMB1 overexpression significantly reduced the activation of IFNβ and ISRE reporter cells in HEK293T-TLR3 cells treated with high-molecular-weight (HMW) Poly I:C (TLR3 agonist). Silencing of *PSMB1* promoted the HMW Poly I:C induced IFNβ activation (Figure 3e). Furthermore, PSMB1 inhibited the TLR3 signaling adaptor TRIF-induced IFNβ, ISRE, and NF-κB promoter activation (Figure 3f). Together, these data suggest that PSMB1 is a negative regulator for the RLR and TLR signaling pathway.

### 3.4. IKK-ε Is the Target of PSMB1

**Figure 4:**
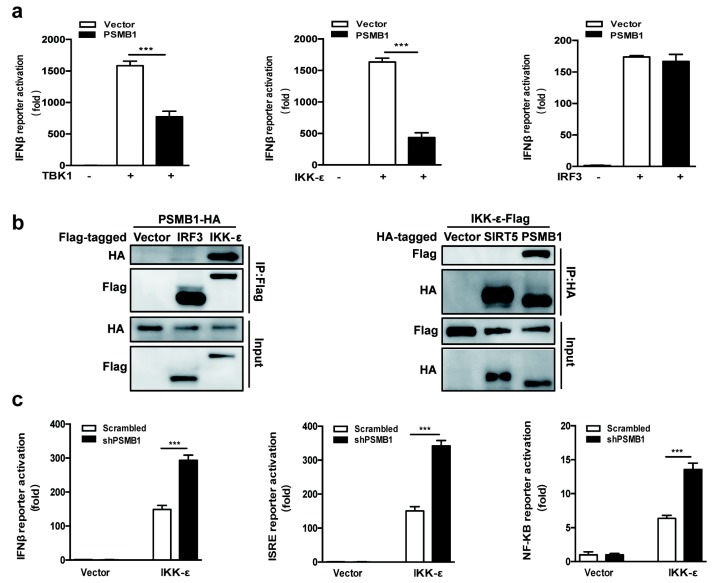
IκB kinase ε (IKK-ε) is the target of PSMB1. (**a**) PSMB1 inhibits RLR and TLR3-mediated IFN signaling. Quantification of IFNβ promoter activity of HEK293T cells expressing empty vector or PSMB1 together with TBK1, IKK-ε, or IRF3, respectively. (**b**) PSMB1 interacts with IKK-ε. Co-immunoprecipitation (Co-IP) of PSMB1 and IKK-ε from HEK293T cells expressing Flag-IKK-ε and HA-PSMB1 using anti-Flag or anti-HA antibody pull-down, followed by immunoblotting. Flag-IRF3 and HA-SIRT5, were used as a negative control. (**c**) Quantification of IFNβ, ISRE, and NF-κB promoter activities in scrambled shRNA or *PSMB1* shRNA-treated HEK293T cells transfected with either empty vector or Flag-IKK-ε plasmid. Data shown are the mean ± SEM; *** *p* < 0.001. Representative results are from at least three independent experiments.

To identify the potential target of PSMB1, we assessed the effects of PSMB1 on IFNβ activation in HEK293T cells mediated by the molecules in the antiviral signaling pathway. We overexpressed the key molecules from the Rig-I and TLR3 signaling pathway together with PSMB1 in HEK293T cells. The reporter assay showed that PSMB1 inhibited TBK1-mediated and IKK-ε-mediated but not downstream transcription factor IRF3-mediated activation of the IFNβ promoter (Figure 4a). These data suggest that PSMB1 might target IKK-ε.

To confirm that PSMB1 targets IKK-ε, we investigated their interaction by using co-immunoprecipitation (Co-IP). PSMB1 plasmid with HA tag and IKK-ε plasmid with Flag tag were transfected into HEK293T cells. The coimmunoprecipitation experiments in HEK293T cells showed that PSMB1 is associated with IKK-ε (Figure 4b). 

We further overexpressed IKK-ε in control or *PSMB1*-silenced cells and detected the IFNβ, ISRE and NF-κB promoter activation. We found that *PSMB1* silencing promoted the IKK-ε-induced antiviral signaling activation (Figure 4c). Together, these data demonstrate that PSMB1 interacts with IKK-ε and negatively regulates the RNA virus-induced IFNβ and NF-κB activation.

### 3.5. PSMB1 Promotes IKK-ε Degradation 

**Figure 5:**
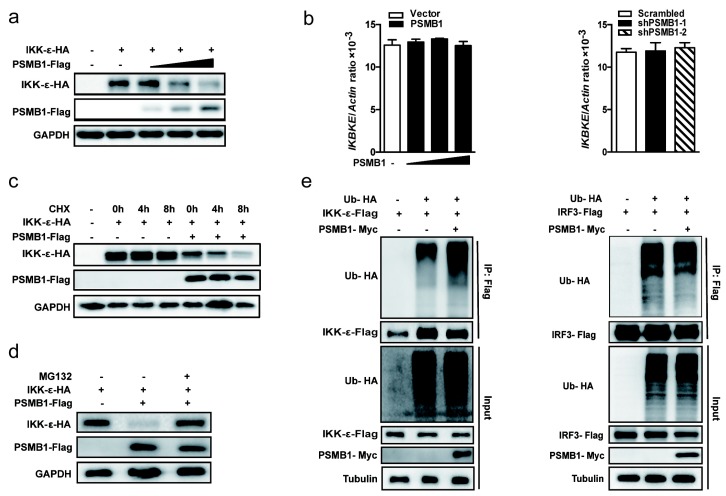
PSMB1 promotes IKK-ε degradation. (**a**) Quantification of IKK-ε protein level by immunoblotting from HEK293T cells expressing increasing amounts of Flag-PSMB1 together with HA-IKK-ε. (**b**) Quantification of the *IKBKE* mRNA level by qPCR in HEK293T cells expressing increasing amounts of PSMB1 (left panel) or *PSMB1*-targeting shRNA (right panel). Data shown are the mean ± SEM. (**c**) Quantification of the IKK-ε protein level by immunoblotting from HEK293T cells transfected with the indicated plasmids and treated with 75 ng/mL of cycloheximide (CHX) for the indicated time points. (**d**) Quantification of IKK-ε protein level by immunoblotting from HEK293T cells transfected indicated plasmids and treated with 10 uM MG132 for 6 h. (**e**) HEK293T cells were co-transfected with HA-ubiquitin (HA-Ub), Myc-PSMB1, Flag-IKK-ε or Flag-IRF3. Cells were treated with 10 uM MG132 for 6 h. Anti-Flag IKK-ε or IRF3 immunoprecipitates were analyzed by immunoblotting with anti-HA antibody. Representative results are from at least three independent experiments.

Since PSMB1 is the component of proteasome complex and interacts with IKK-ε, we examined whether PSMB1 affects the stability of IKK-ε. We transfected HEK293T cells with expression plasmids encoding IKK-ε together with increasing amounts of PSMB1. PSMB1 overexpression decreased the protein level of IKK-ε in a dose-dependent manner (Figure 5a). However, the mRNA level of endogenous *IKBKE* remained unchanged by overexpression of PSMB1 or knockdown of *PSMB1* (Figure 5b). Furthermore, PSMB1 overexpression significantly promoted protein degradation of IKK-ε in cycloheximide (CHX) chase experiments (Figure 5c). These results indicate that PSMB1 regulates IKK-ε expression at the post-transcriptional level. 

To further investigate the PSMB1-mediated inhibition of IKK-ε expression, HEK293T cells were co-transfected with plasmids expressing PSMB1 and IKK-ε together with the proteasome inhibitor MG132. PSMB1-mediated IKK-ε degradation could be blocked by MG132 (Figure 5d). We next examined the effects of PSMB1 on IKK-ε ubiquitination. IKK-ε ubiquitination was significantly enhanced upon PSMB1 overexpression, while PSMB1 did not increase the ubiquitination of IRF3 (Figure 5e). Collectively, these data suggest that PSMB1 promotes IKK-ε degradation through the ubiquitin-proteasome pathway.

## 4. Discussion

PSMB family subunits, the components of the proteasome complex, have been demonstrated to play an important role in the immune system [20]. PSMB9 deficiency leads to alterations of T-cell repertoire formation in mice [21]. The inhibition of PSMB8 reduces the production of inflammatory cytokines and attenuates the progression of experimental arthritis [22]. However, the involvement of PSMB family subunits in cellular antiviral responses remains unclear. In this study, we found that the expression level of *PSMB1* was upregulated during viral infection. *PSMB1* knockdown attenuated viral replication in HEK293T cells. We further investigated the role of PSMB1 in innate immune responses. Overexpression of PSMB1 significantly inhibited the virus-induced IFNβ promoter activation, whereas silencing of *PSMB1* increased the production of IFNβ. These findings suggest that PSMB1 is a negative regulator of virus-induced innate immune responses.

Excessive production of type I interferons has been linked to a pathologic role during host responses to viral infection and must be tightly regulated [23]. The negative regulators of type I IFN can target the innate sensors and downstream signaling molecules through direct interaction or post-translational modification [24]. NLRX1 (a member of the NLR family) has been shown to be a negative regulator of IFN induction by preventing the interaction between RIG-I and MAVS [25]. Smurf2 negatively regulates IFN production by K48 ubiquitination of MAVS for degradation [26]. FBXO17 suppresses type I IFN signaling by recruiting phosphatase 2a (PP2A) for downstream transcription factor IRF3 dephosphorylation and deactivation [27]. IKK-ε is a central component of the type I IFN signaling pathway [28]. A20 and CYLD have been reported to control the activity of IKK-ε. The ubiquitin-editing enzyme A20 cooperates with the adaptor molecule TAX1BP1 to terminate IFN signaling by disrupting Lys63-linked polyubiquitination of IKK-ε [29]. CYLD is a deubiquitinase, which physically interacts with RIG-I and negatively regulates RIG-I ubiquitination, thereby inhibiting RIG-I-mediated activation of IKK-ε [30]. In this study, several lines of evidence indicated that PSMB1 targets IKK-ε to inhibit IFNβ production. First, overexpression of PSMB1 inhibited IKK-ε-, but not the downstream transcription factor IRF3-mediated activation of IFNβ promoter. Second, silencing of PSMB1 promoted the IKK-ε- induced IFNβ activation. Third, PSMB1 directly interacted with IKK-ε. Together our results demonstrate that PSMB1 negatively regulates the RNA virus-induced IFNβ activation by targeting IKK-ε.

Proteasomal degradation of RIG-I and downstream signaling molecules through ubiquitin-proteasome system has been reported to prevent excessive RLR signaling [31]. PSMA7, a subunit of the 20S proteasome complex, has been shown to be a negative regulator of antiviral signaling. PSMA7 interacts with the RIG-I adaptor molecule MAVS and recruits MAVS to the proteasome complex for degradation [32]. The E3-ligase RNF125 is responsible for the attachment of K48-linked polyubiquitin chains to RIG-I, leading to the degradation of RIG-I and thereby inhibiting RLR signaling [33]. MAVS ubiquitination by TRIM25 and degradation by the proteasome is involved in the production of type I interferon [34]. GP73 represses host innate immunity by interacting with TRAF6 and promoting TRAF6 degradation [35]. Triad3A is reported to negatively regulate the RIG-I pathway through Lys48-linked, ubiquitin-mediated degradation of the RIG-I signaling component TRAF3 [36]. Our study has demonstrated that PSMB1 is involved in the stability of IKK-ε. The overexpression of PSMB1 promoted the ubiquitination and proteasomal degradation of IKK-ε.

Transferring substrates to the proteasome complex is a key step for proteasomal degradation. The proteasome subunit PSMA7 was found to interact specifically with HIF-1α and transfer HIF-1α for proteasome-dependent degradation [37]. PSMB1 is reported to interact with the oncogenic protein BCL-3 and to recruit BCL-3 to the proteasome complex [38]. Our study demonstrated that PSMB1 interacts with IKK-ε and facilitates proteasomal degradation of IKK-ε. These results suggest that PSMB1 may have a role in recruiting substrates to the proteasome complex. As non-catalytic β-subunit, PSMB1 has been proposed to contribute to the assembly and stability of the 20S proteasomes and establish the proteolytic environment [39,40]. Further studies are required to elucidate the detailed mechanism for PSMB1-mediated regulation of proteasome activity. 

In conclusion, our study identified PSMB1 as a critical negative regulator of RIG-I- and TLR3-mediated cytokine production. Mechanistically, PSMB1 targets the signaling molecule IKK-ε, promoting proteasomal degradation of IKK-ε. An improved understanding of this regulation mechanism will provide potential strategies against infectious and inflammatory disorders.
